# Association between preoperative hemoglobin and postoperative moderate and severe anemia among patients undergoing primary total knee arthroplasty: a single-center retrospective study

**DOI:** 10.1186/s13018-021-02727-5

**Published:** 2021-09-26

**Authors:** Guorui Cao, Xiuli Yang, Hong Xu, Chen Yue, Zeyu Huang, Shaoyun Zhang, Songtao Quan, Junna Yao, Minglu Yang, Fuxing Pei

**Affiliations:** 1 Department of Knee Injury (2), Luoyang Orthopedic Hospital of Henan Province, Orthopedic Hospital of Henan Province, Luoyang, 471000 Henan Province People’s Republic of China; 2 Department of Orthopaedic Surgery, West China Hospital, Sichuan University, No. 87 Guoxue Road, Chengdu, 610041 Sichuan Province People’s Republic of China; 3 Department of Orthopedics, The Third Hospital of Mianyang, Sichuan Mental Health Center, Mianyang, 621000 Sichuan Province People’s Republic of China

**Keywords:** Moderate and severe anemia, Risk factors, Total knee arthroplasty, Preoperative hemoglobin

## Abstract

**Background:**

Postoperative moderate and severe anemia (PMSA) has been a serious perioperative complication in primary total knee arthroplasty (TKA). However, the ideal cutoff values to predict PMSA is still undetermined. The aim of this study was (1) to identify the risk factors associated with PMSA and (2) to establish the cutoff values of preoperative hemoglobin (HB) associated with increased PMSA in primary TKA.

**Methods:**

We identified 474 patients undergoing primary TKA and separated those in which PMSA (HB was less than 110 g/L on postoperative day 1 and 3) was developed from those without PMSA. Multivariate logistic regression model was used to identify independent risk factors for PMSA. Area under the receiver-operator curve (AUC) was used to determine the best-supported preoperative HB cutoff across all the patients.

**Results:**

The PMSA rate in primary TKA was 53.2%. Significant risk factors were lower preoperative HB (OR [odds ratio] = 1.138, 95% CI [confidence interval] = 1.107–1.170, *p* < 0.001) and more intraoperative blood loss (OR = 1.022, 95% CI 1.484–4.598, *p* < 0.001).

A preoperative HB cutoff value that maximized the AUC was 138.5 g/L for men (sensitivity: 79.4%, specificity: 75.0%) and 131.5 g/L for women (sensitivity: 74.7%, specificity: 80.5%), respectively.

**Conclusion:**

We should recognize and consider the related risk factors to establish specific, personalized risk assessment for PMSA, including preoperative HB and intraoperative blood loss. Of these, preoperative HB was a referable tool to predict PMSA in primary TKA.

## Introduction

Total knee arthroplasty (TKA) is an effective treatment to correct deformity, relieve pain and improve quality of life for advanced osteoarthritis of the knee [1]. Although the application of enhanced recovery protocols, especially the use of tranexamic acid (TXA), dramatically reduced perioperative blood loss and transfusion following primary TKA, postoperative moderate and severe anemia (PMSA) has been still a frequent and troublesome issue [2, 3]. PMSA was always associated with longer length of stay (LOS), higher readmission risk and increased risk of complications and morbidity, such as transfusion, joint infection, bleeding events, kidney injury and even death [4–7]. Given the increasing number of TKAs performed around the world, an important concern for surgeons was how to minimize the risk of PMSA in primary TKA [8].

To our best knowledge, no previous study has identified the specific risk factors associated with PMSA for primary TKA. Considering that preoperative hemoglobin (HB) was a strong predictor for transfusion after TKA, one possible predictor for PMSA was preoperative HB [9, 10]. Currently, the risk factor for PMSA, the sensitive and specific preoperative HB value for predicting PMSA following primary TKA have not been determined. Therefore, we perform a retrospective cohort study to investigate (1) what are the risk factors associated with PMSA? (2) If preoperative HB is highly associated with PMSA, what is appropriate cutoff values of preoperative HB for PMSA in primary TKA?

## Materials and methods

### Patients and design

This study was a single-center retrospective cohort study and approved by the hospital’s Institutional Review Board. The patients undergoing primary TKA owing to osteoarthritis and rheumatoid arthritis were included from October 2015 to August 2018 in our institution. We excluded the patients with any of the following situations: cardiovascular problems, history of deep venous thrombosis (DVT) or pulmonary embolism (PE) and incomplete medical records.

The blood samples were tested preoperatively, on postoperative (POD) 1 and POD3. Moderate anemia was defined as a HB level between 80 and 110 g/L and severe anemia was defined as a HB level < 80 g/L for both genders [11]. Because of small number of severe anemia (*n* = 5), patients with moderate and severe anemia were analyzed together. The enrolled patients were divided into PMSA and non-PMSA groups according to level of postoperative hemoglobin (HB) on POD1 or POD3.

### Surgical technique

All the surgeries were conducted by the same senior surgeon under general anesthesia. The operations were performed through a midline skin incision, medial parapatellar approach and a measured resection technique. The prosthesis was cemented posterior-stabilized prosthetic design with patellar resurfacing. Controlled hypotension was induced with the blood pressure being maintained 90–110 mmHg/60–70 mmHg during operation. No tourniquet, nerve block, intravenous patient-controlled analgesia or blood salvage system were used.

### Perioperative protocol

All the patients received enhanced recovery protocols [12]. The physical prophylaxis and chemoprophylaxis were applied to prevent DVT and PE. Physical prophylaxis included the exercises of ankle pump and knee extension, and the application of intermittent pneumatic compression device early postoperatively. Chemoprophylaxis included the application of low-molecular-weight heparin (2000 IU in 0.2 ml; Clexane, Sanofi-Aventis, France) or 10 mg Rivaroxaban (Xarelto, Bayer, Germany) 6–8 h postoperatively, repeating once a day for 14 days. Doppler ultrasound examinations were used routinely to detect DVT preoperatively, on POD3 and POD14.

The blood management strategies were implemented on the basis of our previous studies [13, 14]. The TXA (20 mg/kg) was administered intravenously 5–10 min before skin incision, along with 1 g intravenous TXA (diluted in 100 mL of normal saline solution) at 3, 6, 12 and 24 h postoperatively. Patients received the combination of 10,000 IU of erythropoietin subcutaneously once a day for 5 days and 200 mg of intravenous iron supplements once every other day when the perioperative HB was less than 110 g/L, and only 300 mg of oral iron supplements every day when the level of Hb was 110–130 g/L for males and 110–120 g/L for females. Transfusions were applied when the HB value was < 70 g/L or 70–100 g/L with symptoms of anemia, such as altered mental status, palpitations or decreased exercise tolerance [15].

### Outcome measurements

Demographic characteristics and comorbidities, including age, gender, diagnosis, body mass index, incidence of hypertension, diabetes, coronary heart disease and so on were collected. Laboratory markers (Hb, hematocrit (Hct), erythrocyte sedimentation rate, C-reactive protein and interleukin 6) were tested preoperatively, on POD1 and POD3. In addition, American Society of Anesthesiologists physical status classification, the application of TXA and drainage, operative time, intraoperative blood loss, allogenic transfusion units and rate, length of stay (LOS) after surgery, total expenses and postoperative complications were carefully recorded. Wound complication were defined and classified on the basis of previous study [16].

### Statistical analyses

All analyses were performed using SPSS version 22.0 (SPSS Inc., USA) and EmpowerStats (http://www.empowerstats.com/). Descriptive and univariate analyses were initially conducted using SPSS software. Excluding variables with significant correlation, statistically significant or close to statistically significant variables were enrolled a multivariable logistic regression model to examine independent risk factor for PMSA in primary TKA. Receiver operating characteristic (ROC) curve and curve fitting were performed by EmpowerStats software. Youden’s index was applied to determine the optimal predictive cutoffs for preoperative HB. Independent *t*-test was used for continuous variables and chi-squared test or Fisher's exact test for categorical and dichotomous variables. A *p* value of < 0.05 was considered significant.

## Results

**Fig. 1:**
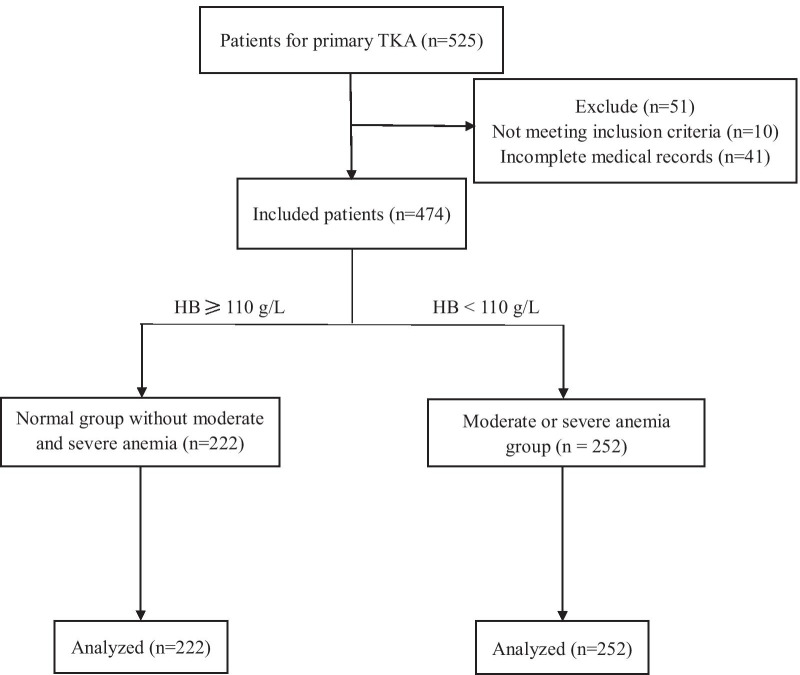
Flow diagram of patients shows the study design

Five hundred and twenty-five patients were included initially. Fifty-one patients were excluded because of incomplete medical records or inconformity to inclusion criteria. Eventually, 474 patients were enrolled, including 252 patients with PMSA and 222 patients without PMSA (Fig. 1). Totally, the prevalence of PMSA was 53.2% for primary TKA, 12 cases (12/252, 5.2%) developed anemia on POD1 and 239 cases (239/252, 94.8%) on POD3. In addition, only 5 (5/252, 2.0%) cases were severe anemia which was entirely occurred on POD3 and other were moderate anemia.

Baseline characteristics of PMSA and non-PMSA patients are shown in Table 1. The risk factors for PMSA after univariate analyses were female, lower body mass index (BMI), diagnosis as rheumatoid arthritis, preoperative anemia, lower level of preoperative HB and Hct, more intraoperative blood loss. Besides, the application of TXA was a protective factor (*p* < 0.05).

**Table 1:** Baseline of patients with moderate and severe anemia after primary TKA

Baseline characteristic	Non-DMSA group, *N* = 222	DMSA group, *N* = 252	*p*
*Demographic characteristics*			
Age($\overline{X }$±*S*)	66.8 ± 7.8	66.2 ± 7.9	0.484
Female (*N*, %)	154 (69.4%)	236 (93.7%)	< 0.001*
BMI($\overline{X }$±S, kg/m^2^)	26.4 ± 3.5	25.4 ± 3.7	0.003*
Diagnosis (RA/OA)	1/221	12/240	0.004*
Preoperative knee ROM (°)	99.0 ± 17.2	100.0 ± 15.1	0.480
Preoperative HSS	49.9 ± 8.3	48.9 ± 8.4	0.275
*Comorbidities (N, %)*			
Hypertension	113 (50.9%)	138 (54.8%)	0.401
Diabetes	31 (14.0%)	41 (16.3%)	0.485
Coronary heart disease	15 (6.8%)	16 (6.3%)	0.858
Preoperative anemia	6 (6.8%)	46 (18.3%)	< 0.001*
*Preoperative laboratories*			
HB (g/L)	140.2 ± 11.0	125.1 ± 10.2	< 0.001*
Hct	42.0 ± 3.1	38.3 ± 3.1	< 0.001*
ESR (mm/h)	21.3 ± 20.2	22.4 ± 17.8	0.116
CRP (mg/L)	3.50 ± 2.77	3.77 ± 3.67	0.372
IL-6 (mg/L)	3.8 ± 2.4	4.3 ± 4.2	0.126
*Operative variables*			
ASA class (*N*, %)			0.959
1–2	174 (78.4%)	198 (78.6%)	
≥ 3	48 (21.6%)	54 (21.4%)	
Drainage use (*N*, %)	42 (18.9%)	66 (26.2%)	0.060
TXA use (*N*, %)	217 (97.7%)	226 (89.7%)	< 0.001*
Operative time (min)	76.4 ± 12.1	77.7 ± 15.5	0.340
Intraoperative blood loss (mL)	110.0 ± 22.8	130.9 ± 34.7	< 0.001*

BMI, body mass index; RA, rheumatoid arthritis; OA, osteoarthritis; ROM, range of motion; HSS, hospital for special surgery knee score; HB, hemoglobin; Hct, hematocrit; ESR, erythrocyte sedimentation rate; CRP, C reaction protein; IL-6, interleukin-6; ASA, American Society of Anesthesiologists; TXA, Tranexamic acid

*p* value calculated using independent *t*-test, Pearson's chi-squared test or Fisher's exact test

^*^Significant difference

Risk factors independently associated with PMSA after logistic regression included more intraoperative blood loss (OR [odds ratio] = 1.022, 95% CI [confidence interval]) = 1.484–4.598, *p* < 0.001) and lower preoperative Hb (OR = 1.138, 95% CI 1.107–1.170, *p* < 0.001) (Table 2).

**Table 2:** Logistic regression models for moderate and severe anemia after primary TKA

Parameter	Odds ratio	95% Confidence interval	*p*
Female	1.931	0.928–4.015	0.078
BMI	1.033	0.967–1.104	0.340
Diagnosis, RA	3.171	0.353–28.456	0.303
Preoperative Hb (g/L)	1.138	1.107–1.170	< 0.001*
Drainage use	1.354	0.734–2.499	0.333
TXA use	0.358	0.085–1.506	0.161
Intraoperative blood loss	1.022	1.010–1.034	< 0.001*

BMI, body mass index; RA, rheumatoid arthritis; Hb, hemoglobin; TXA, Tranexamic acid

*p* value calculated using multivariate logistic regression

^*^Significant difference

**Fig. 2:**
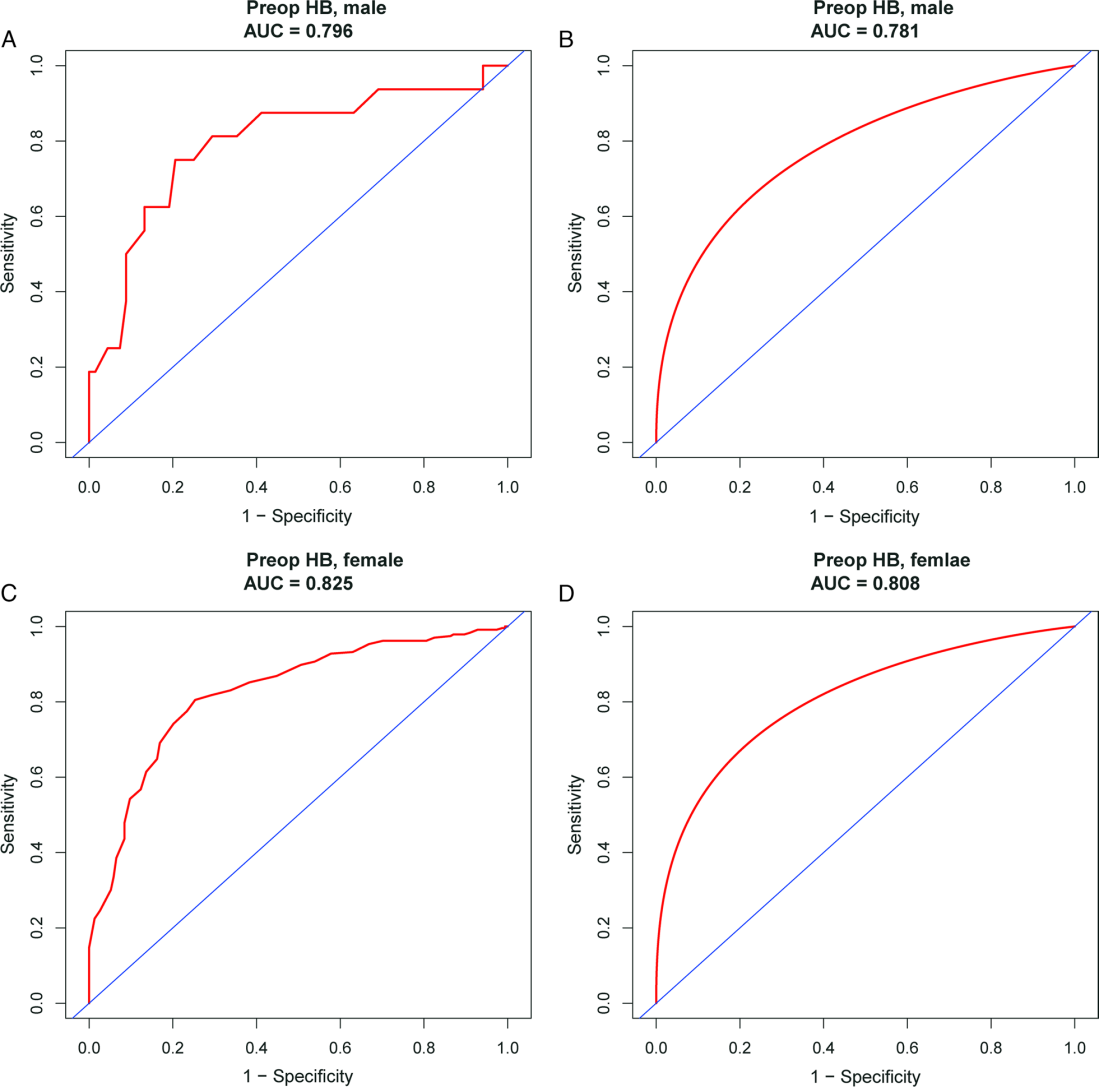
The ROC curve for predicting PMSA using preoperative HB for male and female

**Fig. 3:**
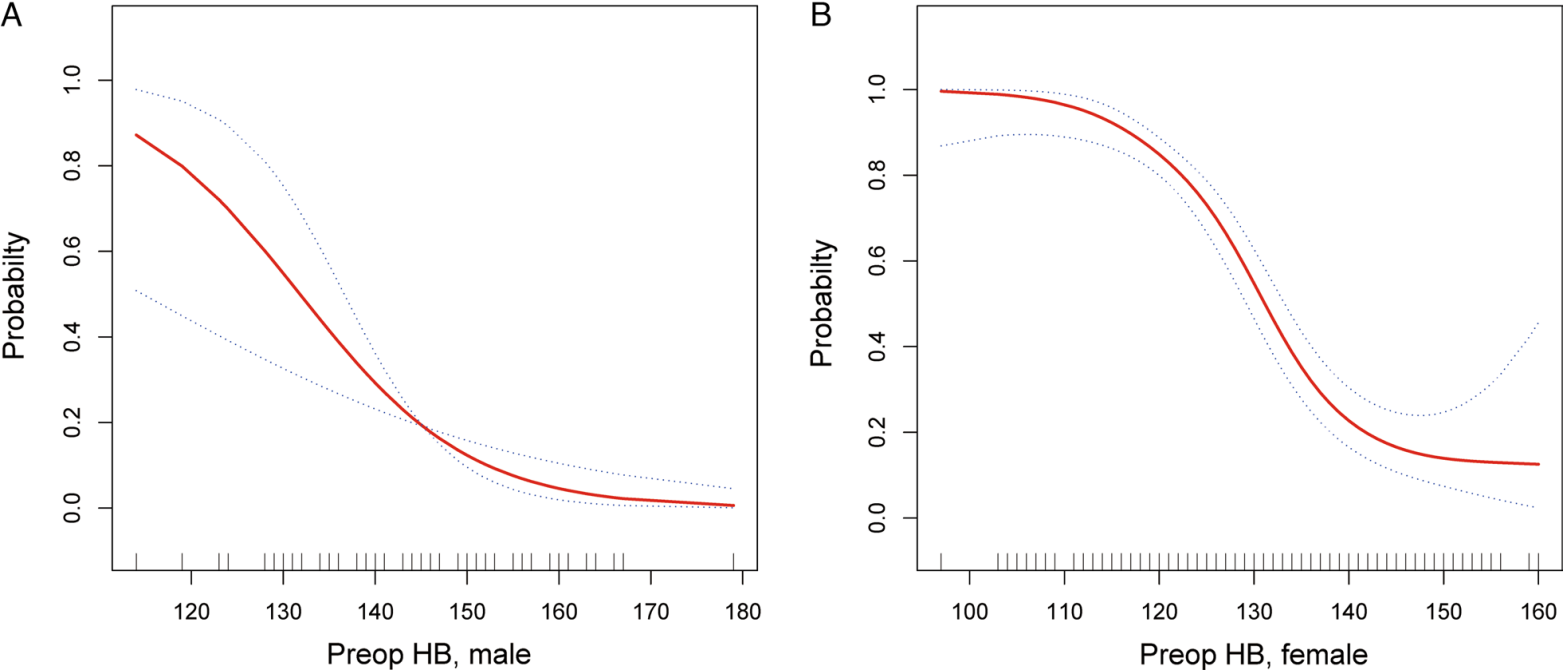
The predictive probability of PMSA by preoperative HB adjusted for sex

Preoperative HB was found to be the variable most highly associated with PMSA for men (AUC, 0.796) and women (AUC, 0.825), respectively. Moreover, the value of AUC following bootstrap smooth ROC was 0.781 and 0.808 separately (Fig. 2). The optimal predictive cutoff of preoperative HB for PMSA was 138.5 g/L (sensitivity: 79.4%, specificity: 75.0%) in male and 131.5 g/L (sensitivity: 74.7%, specificity: 80.5%) in female sex (Table 3). As expected, the risk of PMSA in primary TKA decreased when the preoperative HB increase for men and women (Fig. 3).

**Table 3:** The diagnostic value of preoperative HB for PMSA

Variables	Youden's index	Preoperative		Maximize	
		Cutoff	AUC	Sensitivity (%)	Specificity (%)
Male	0.544	138.5 (g/L)	0.796/0.781	79.4	75.0
Female	0.552	131.5 (g/L)	0.825/0.808	74.7	80.5

HB, hemoglobin; PMSA, postoperative moderate or severe anemia; AUC, Area under the receiver operating characteristics curve

The situations of LOS, expenses and related complication are summarized in Table 4. The LOS in PMSA group was longer than that in non-PMSA group (3.92 ± 0.94 vs 3.06 ± 0.92, *p* = 0.002). The similar trend was shown in hospitalization expenses (59,532.3 ± 9618.2 vs 57,143.1 ± 9246.5, *p* = 0.031). In addition, the incidence of complications, including transfusion, DVT, PE, wound complications and so on did not reach statistical difference between the two groups (*p* > 0.05).

**Table 4:** LOS, expenses and complications

Variables	Non-DMSA group, *N* = 222	DMSA group, *N* = 252	*p*
LOS	3.06 ± 0.92	3.92 ± 0.94	0.002*
Hospitalization expenses ∆	57,143.1 ± 9246.5	59,532.3 ± 9618.2	0.031*
Transfusion	0	2 (0.8%)	0.501
Death	0	0	–
DVT	2 (0.9%)	1(0.4%)	0.602
PE	0	0	–
Nausea and vomiting	17 (7.7%)	29 (11.5%)	0.158
Cardiac infarction	1 (0.5%)	0	0.468
Stroke	0	0	–
Acute renal failure	0	0	–
Wound complications	23 (10.4%)	29 (11.5%)	0.690
Readmission	2 (0.9%)	2 (0.8%)	1.000

Wound complications included exudation, bleeding, swelling, infection and impaired wound healing

LOS, length of stay; DVT, deep venous thrombosis; PE, pulmonary embolism

*p* value calculated using independent *t*-test, Pearson's chi-squared test or Fisher's exact test

∆ Results were presented as Chinese yuan

^*^Significant difference

## Discussion

Anemia was a common complication after orthopedic surgery, resulting in a series of problems. Generally, mild anemia could be corrected by oral iron supplements, reinforced nutrition. By comparison, PMSA was usually difficult to resolve, needing further treatments, such as intravenous iron, erythropoietin, even transfusion [17, 18]. Moreover, patients who developed PMSA had higher risk of developing postoperative complications relative to patients with mild anemia following joint replacement [19]. Thus, we selected PMSA as the outcome variable, evaluating related risk factor and attempting to decrease the rate.

Current literature is mainly limited to describe the effect of preoperative anemia on the transfusion, LOS, complication, functional and quality of life outcomes in the setting of primary TKA [19–21]. As preoperative anemia, postoperative anemia is also an independent risk factor for delayed postoperative rehabilitation, increasing complications and readmission rates [4, 5, 7]. Our study showed that preoperative HB and intraoperative blood loss are significantly associated with increased PMSA. The ideal preoperative HB cutoff value was 131.5 g/L for males and 138.5 g/L for females in primary TKA. Identifying a robust preoperative HB cutoff could offer opportunities for doctors to treatment patient’s anemia and decrease the PMSA risk. Patients whose preoperative HB was less than the cutoff value could receive intervention early, such as erythropoietin and iron supplementation, to avoid PMSA and associated complications [22–27].

Currently, there is a lack of consensus and evidence-based practice when it comes to the risk factors for PMSA in patients undergoing TKA. However, the risk factor for transfusion in TKA was extensively studied. The researchers concluded that lower level of preoperative HB and increased amount of intraoperative blood loss were independently associated with transfusion in TKA [28–30]. Considering the trigger of transfusion and diagnosis of PMSA was both mainly decided by the level of postoperative HB, the risk factor for transfusion and PMSA in primary TKA could be concordant. Our study confirmed that risk factors for PMSA in primary TKA were lower preoperative HB and more intraoperative blood loss.

Previous studies have used other methods in evaluating the cutoffs of preoperative HB to predict transfusion in TKA, but the cutoffs to optimal predict PMSA remained poorly understood. Maempel et al. identified threshold levels of 13.75 g/dL for males and 12.75 g/dl for females. The transfusion and complication rate increased significantly when preoperative was less than the threshold [9]. Yeh et al. found that preoperative HB cutoff values of 12.4 g/dL for age above 70 and 12.1 g/dL for age below 70 could be used to predict postoperative transfusion risk in TKA [10]. In another study, the authors showed that a preoperative HB value of 12.5 g/dL was identified as the optimal cutoff for predicting postoperative transfusion across all patients with the administration of TXA [31]. Because the majority PMSA was moderate anemia in our study and preoperative HB threshold for moderate anemia (80–109 g/L) was higher than that for transfusion (< 70 or 70–100 g/L) [11, 15], the reasonable hypothesis was that preoperative HB cutoff value to predict PMSA should be higher. Our study demonstrated the relationship between preoperative HB and PMSA firstly, finding that131.5 g/L for males and 138.5 g/L for females were essential cutoff value in primary TKA in Chinese population. We provided an early warning value of preoperative HB for surgeons and patients.

In this study, we found that PMSA could lead to prolonged LOS and more expenses, which was consistent with previous studies. Abdullah et al. indicated that anemia was associated with prolonged LOS and increased perioperative transfusion [21]. Another study conducted by Kunz and his colleagues came to a similar conclusion [7]. In addition, substantial researches demonstrated that perioperative anemia could contribute to higher incidence of complication [5–7]. But the incidence of complications between PMSA and non-PMSA patients was comparable in our study. Probable seasons were as follow: firstly, the follow-up period was only 30 days, which was too short to monitor complete complications; secondly, the application of enhanced recovery protocols reduced the complication risk, decreasing the significance between the two groups [12]. Moreover, no patients needed blood transfusion in our study, indicating enhanced recovery protocols, particularly blood management strategies, played an important and beneficial role [3].

There are some limitations in this study. The amount of male cases is too small, only 84 patients (68 in non-PMSA and 26 in PMSA group), reducing the accuracy to identify the risk factors and predict preoperative HB cutoff value for PMSA. In addition, because most patients are discharged from hospital on POD3 and the blood samples are tested on POD1 and 3, we select the HB on POD1 and 3 as the diagnostic node for PMSA. It was reported that postoperative HB may continue to decline [32]. Thus, the incidence of PMSA is not accurate and we fail to monitor the comprehensive prevalence of anemia after primary TKA. Moreover, the follow-up time is only 30 days, leading to inability to evaluate the complications entirely, especially long-term complications, such as deep infection and impaired wound healing. At last, the limitation lies in its retrospective nature and the level of evidence is low. Further studies are requisite. We will conduct a randomized controlled study with longer follow-up time to investigate the relationship of preoperative HB and PMSA following joint arthroplasty in the near future.

## Conclusion

In summary, lower preoperative HB and more intraoperative blood loss are independent risk factors for PMSA. Preoperative HB is a referable tool to predict PMSA. We provide an early warning value of preoperative HB for surgeons and patients undergoing primary TKA.

## Abbreviations

TKA: Total knee arthroplasty
PMSA: Postoperative moderate and severe anemia
HB: Hemoglobin
AUC: Area under the receiver-operator curve
OR: Odds ratio
95% CI: Confidence interval
TXA: Tranexamic acid
LOS: Longer length of stay
DVT: Deep venous thrombosis
PE: Pulmonary embolism
POD1: Postoperative day 1
POD3: Postoperative day 3
BMI: Body mass index

## References

[1] Kurtz S, Ong K, Lau E (2007). Projections of primary and revision hip and knee arthroplasty in the United States from 2005 to 2030. J Bone Joint Surg Am.

[2] Varghese VD, Liu D, Ngo D (2021). Efficacy and cost-effectiveness of universal pre-operative iron studies in total hip and knee arthroplasty. J Orthop Surg Res.

[3] Wang D, Wang HY, Luo ZY (2018). Blood-conserving efficacy of multiple doses of oral tranexamic acid associated with an enhanced-recovery programme in primary total knee arthroplasty: a randomized controlled trial. Bone Joint J.

[4] Bailey A, Eisen I, Palmer A (2021). Preoperative anemia in primary arthroplasty patients-prevalence, influence on outcome, and the effect of treatment. J Arthroplast.

[5] Enokiya T, Hasegawa M, Morikawa Y (2020). Postoperative anaemia is a risk factor for bleeding-related event in thromboprophylaxis using fondaparinux sodium injection after total knee or hip arthroplasty. Biol Pharm Bull.

[6] Koch CG, Li L, Sun Z (2017). Magnitude of anemia at discharge increases 30-day hospital readmissions. J Patient Saf.

[7] Kunz JV, Spies CD, Bichmann A (2020). Postoperative anaemia might be a risk factor for postoperative delirium and prolonged hospital stay: A secondary analysis of a prospective cohort study. PLoS ONE.

[8] Maradit Kremers H, Larson DR, Crowson CS (2015). Prevalence of total hip and knee replacement in the United States. J Bone Joint Surg Am.

[9] Maempel JF, Wickramasinghe NR, Clement ND (2016). The pre-operative levels of haemoglobin in the blood can be used to predict the risk of allogenic blood transfusion after total knee arthroplasty. Bone Joint J.

[10] Yeh JZ, Chen JY, Bin Abd-Razak HR (2016). Preoperative haemoglobin cut-off values for the prediction of post-operative transfusion in total knee arthroplasty. Knee Surg Sport Traumatol Arthrosc.

[11] [Anonymous]. Nutritional anaemias. Report of a WHO scientific group. World Health Organization technical report series 1968; 405:5–37. 4975372

[12] Ripolles-Melchor J, Abad-Motos A, Diez-Remesal Y (2020). JAMA Surg.

[13] Cao G, Huang Q, Xu B (2017). Multimodal nutritional management in primary total knee arthroplasty: a randomized controlled trial. J Arthroplast.

[14] Zhang S, Huang Q, Xu B (2018). Effectiveness and safety of an optimized blood management program in total hip and knee arthroplasty: a large, single-center, retrospective study. Medicine.

[15] Xie J, Ma J, Yao H (2016). Multiple boluses of intravenous tranexamic acid to reduce hidden blood loss after primary total knee arthroplasty without tourniquet: a randomized clinical trial. J Arthroplast.

[16] Dennis DA (2002). Wound complications in TKA. Orthopedics.

[17] Lasocki S, Krauspe R, von Heymann C (2015). PREPARE: the prevalence of perioperative anaemia and need for patient blood management in elective orthopaedic surgery: a multicentre, observational study. Eur J Anaesth.

[18] Lee QJ, Mak WP, Yeung ST (2015). Blood management protocol for total knee arthroplasty to reduce blood wastage and unnecessary transfusion. J Orthop Surg.

[19] Gu A, Malahias MA, Selemon NA (2020). Increased severity of anaemia is associated with 30-day complications following total joint replacement. Bone Joint J.

[20] Abdullah HR, Ranjakunalan N, Yeo W (2019). Association between preoperative anaemia and blood transfusion with long-term functional and quality of life outcomes amongst patients undergoing primary total knee arthroplasty in Singapore: a single-centre retrospective study. Qual Life Res.

[21] Abdullah HR, Sim YE, Hao Y (2017). Association between preoperative anaemia with length of hospital stay among patients undergoing primary total knee arthroplasty in Singapore: a single-centre retrospective study. BMJ Open.

[22] Biboulet P, Motais C, Pencole M (2020). Preoperative erythropoietin within a patient blood management program decreases both blood transfusion and postoperative anemia: a prospective observational study. Transfusion.

[23] Pennestrì F, Maffulli N, Sirtori P (2019). Blood management in fast-track orthopedic surgery: an evidence-based narrative review. J Orthop Surg Res.

[24] Sinclair RC, Duffield KE, de Pennington JH (2020). Improving preoperative haemoglobin using a quality improvement approach to treat iron deficiency anaemia. BMJ Open Qual.

[25] Migliorini F, Maffulli N, Aretini P (2021). Impact of tourniquet during knee arthroplasty: a bayesian network meta-analysis of peri-operative outcomes. Arch Orthop Trauma Surg.

[26] Migliorini F, Maffulli N, Betsch M (2021). Closed suction drainages in Lower Limb Joint Arthroplasty: a level I evidence based meta-analysis. Surgeon.

[27] Roberts M, Ahya R, Greaves M (2000). A one-centre prospective audit of peri- and postoperative blood loss and transfusion practice in patients undergoing hip or knee replacement surgery. Ann R Coll Surg Engl.

[28] Erben HC, Hess F, Welter J (2021). Perioperative blood transfusions in hip and knee arthroplasty: a retrospective assessment of combined risk factors. Arch Orthop Trauma Surg.

[29] Huang Z, Huang C, Xie J (2018). Analysis of a large data set to identify predictors of blood transfusion in primary total hip and knee arthroplasty. Transfusion.

[30] Song K, Pan P, Yao Y (2019). The incidence and risk factors for allogenic blood transfusion in total knee and hip arthroplasty. J Orthop Surg Res.

[31] Ryan SP, Klement MR, Green CL (2019). Preoperative hemoglobin predicts postoperative transfusion despite antifibrinolytics during total knee arthroplasty. Orthopedics.

[32] Gomez-Barrena E, Ortega-Andreu M, Padilla-Eguiluz NG (2014). Topical intra-articular compared with intravenous tranexamic acid to reduce blood loss in primary total knee replacement: a double-blind, randomized, controlled, noninferiority clinical trial. J Bone Joint Surg.

